# “I am like a camel struggling in the desert”: understanding Chinese foreign language teachers’ perceived identities through metaphors

**DOI:** 10.3389/fpsyg.2026.1696296

**Published:** 2026-03-16

**Authors:** Yiqian Yan, Hua Lu, Shihao Ge

**Affiliations:** 1 School of Foreign Languages, Hangzhou Dianzi University, Hangzhou, China; 2 School of Foreign Studies, Anhui Polytechnic University, Wuhu, China; 3 School of Humanities, Tiangong University, Tianjin, China

**Keywords:** China context, foreign language teachers, higher education, identity perception, language teacher identity, metaphor

## Abstract

**Introduction:**

With the radical changes taking place in China’s foreign language education landscape, Chinese foreign language (CFL) teachers encounter unprecedented challenges in their professional lives. In contrast, little is known about their perceived identities. Using metaphor as a lens, this study investigates how CFL teachers metaphorically represent their identities and what factors contribute to their perceptions.

**Methods:**

Data were collected from metaphor elicitation tasks, one-on-one follow-up interviews, and documents. Eighty-two teachers (19 males and 63 females) from four types of universities voluntarily participated in this study.

**Results:**

Findings indicate that the elicited 91 metaphors carry positive, negative, neutral and mixed meanings. While teachers offering positive metaphors (about 56%) mainly describe themselves as guides, facilitators, nurturers, leaders, and light emitters, quite a number of teachers produce a wide range of negative metaphors (about 38.5%), which are categorized into hard workers, laborious animals, service providers, petty beings, and inanimate objects. This study identifies teachers’ personal philosophy and self-efficacy, institutional demands, socio-cultural environment, students’ attitude, disciplinary status, and faculty support as the major contributing factors that affect teachers’ identity perceptions. The findings further reveal that CFL teachers’ mixed identity perceptions are subject to the interplay of multiple factors.

**Implications:**

This study has implications for policymakers and teacher educators in terms of how to improve CFL teachers’ identity perceptions amid the shifting and complex higher education landscape.

## 1 Introduction

Language teacher identity (LTI), which is a teacher’s answer to “who I am as a teacher” (Beijaard et al., 2004), has emerged as a prominent topic in teacher education (Nazari et al., 2025; Uştuk and Hu, 2025; Yan et al., 2024). Previous research has demonstrated that teachers need to negotiate and reconstruct their identities under complex and somewhat unstable circumstances (Seyri and Nazari, 2022), and a robust identity is pivotal to teachers’ wellbeing, performance, as well as continuing professional development (Herrera and Martínez-Alba, 2022; Liu and Sammons, 2022; Lu and Tang, 2025).

LTI researchers used to lament on the lack of an accurate definition of teacher identity (Kılıç and Cinkara, 2019), as teacher identity was considered elusive and difficult to quantify (Beauchamp and Thomas, 2009). With the advent of cognitive psychology, metaphor has proved a useful tool to demystify teacher identity (Lim, 2024; Maddamsetti and Yuan, 2024; Muñoz-Salinas, 2023). While quite a number of studies have offered valuable insights into LTI in different educational contexts through the lens of metaphors (Derakhshan et al., 2023; Kamil, 2022; Thi Nguyen, 2024; Xiong et al., 2015; Yim and Mercer, 2025). It must be noted that most extant LTI studies focus on teachers for young learners in Vietnam (Nguyen, 2016), South Korea (Yim and Mercer, 2025), Iran (Nazari et al., 2023). Given that LTI arises from teachers’ experience and the context in which teachers are situated (Kılıç and Cinkara, 2019), it is worthy of further investigation whether and how LTI varies across different working environments.

### 1.1 Teaching foreign languages in China’s higher education context

China’s higher education (HE) institutions can be categorized into public and private universities. The former are funded by the government, whereas the latter are run by entrepreneurs (Huang et al., 2023). Two striking differences exist in terms of working at public and private universities: First, compared to their counterparts, public university teachers generally enjoy higher salary and a greater sense of job stability. Second, different teacher appraisal systems are adopted. To promote the university ranking, public university leaders usually encourage teachers to publish in high-quality journals, by contrast, to survive in the market and cut down the operation costs, private university leaders tend to assign teachers extra workload, which might distract them from teaching (Wang, 2015).

The Chinese HE system features a hierarchical structure, with elite universities (Project 985, 211 and Double First Class universities) at the top, non-project universities in the middle, and vocational colleges at the bottom (Hu et al., 2017). Universities are different in terms of mission, size, and program types (degree level, degree area, comprehensiveness, and emphasis of programs) (Han et al., 2023). In general, elite universities have priority to admit better students and obtain substantial financial funds (Shu et al., 2020). Given China’s vast territory and the imbalanced regional economic development, teachers working in different cities and regions face differentiated evaluation policies. In general, elite universities, especially those located in first-tier (Beijing, Shanghai, Guangzhou, Shenzhen) or second-tier cities (Tianjin, Hangzhou, Nanjing, etc.) implement more stringent research requirements on teachers compared to those in underdeveloped regions such as the third-tier (provincial capital city) or fourth-tier cities (non-provincial capital city).

CFL teachers are defined as teachers who speak Chinese as the first language and are currently teaching foreign languages (English, Japanese, Russia, etc.) at Chinese universities. In China, English has been taught compulsorily in mainstream education from secondary schools to universities ever since 1978 (Wang, 2015), and Japanese is considered the second largest foreign language (Huang and Feng, 2019). Due to the prominent status of English over other foreign languages, most university students have been studying English for at least 8 years upon admission to university, whereas students majoring in other languages have zero foundation. In many Chinese universities qualified for enrolling English majors, those who teach English majors and those who teach non-English majors are generally assigned to separate Teaching and Research Offices. They teach different courses: while the former teach intensive English, literature, linguistics, translation, etc., the latter teach general English (reading and writing; listening and speaking) courses. However, it’s possible for teachers for English majors to teach non-English majors (when certain teachers apply for sick leave or change of office affiliation). In recent years, however, more and more universities have been canceling English and foreign language majors, and there is a significant reduction in credits and class hours for public foreign language courses at tertiary level (Shu, 2025; Tao, 2019). In addition, following many Chinese universities’ escalated efforts to promote university ranking, language teachers are required to be research active and research productive (He et al., 2025). Other sources of challenges for in-service CFL teachers include the intercultural and linguistic demands inherent in the teaching profession (Zhang and Mohd, 2024), technological advancements (Gao et al., 2024), and China’s language teaching reform policies (Zhang, 2025).

### 1.2 Research aim and questions

Against this backdrop, it remains to be explored how CFL teachers perceive their identities amid the rapidly transforming HE setting. In particular, to the best of our knowledge, no research has involved both public and private universities, and both English teachers and teachers of other foreign languages. Current studies are limited by a small number of participants, as well as the single setting: i.e. either public university, (Gao and Cui, 2021; Wood, 2025; Zhang, 2019), or private tutoring institution (Liu and Sammons, 2022; Xiong et al., 2022). There is little research comparing LTI across different types of universities. This is important given the central role of LTI to language teaching and the stratified Chinese HE system.

We address this dearth of literature by involving English teachers and teachers of other languages from both private and public universities and compares and contrasts their identity perceptions. The findings will deepen our understanding of identity as a dynamic construct, illuminating ways to enhance LTI perceptions in similar settings. The study addresses the following questions:

1. To what extent do CFL teachers’ metaphors suggest their positive and negative perceptions of identities?
2. In which categories can these metaphors be shared in terms of common characteristics?
3. What factors contribute to CFL teachers’ use of particular types of metaphors to represent themselves?

## 2 Literature review

### 2.1 Language teacher identity: definition and influencing factors

Within the LTI literature, there is a consensus over the dynamic, multifaceted, and context-dependent nature of LTI (Derakhshan et al., 2023; Ho, 2025). However, no consensus has been reached on the definition of LTI due to scholars’ varied theoretical perspectives. Some emphasize its discursive and narrative nature (Sherman and Teemant, 2023; Trent, 2020; Yung and Yuan, 2020), some maintain the recognition by others as essential to LTI (Solari and Martín Ortega, 2020; Varghese et al., 2005), while others view future self as an integral component of teacher identity (Van Lankveld et al., 2017; Yan et al., 2024).

Yazan (2018) defined teacher identity as “teachers” dynamic self-conception and imagination of themselves as teachers, which shifts as they participate in varying communities, interact with other individuals, and position themselves (and are positioned by others) in social contexts” (p. 21). In this study, we are not concerned with the future dimension of teacher identity, we focus on teachers’ understanding of who they are rather than other people’s perceptions. Therefore, we draw on Yanzan’s definition and reconceptualize LTI as “a language teacher’s dynamic self-conception of himself/herself as a teacher within his or her situated environment.” This is the theoretical stance we adopt in understanding LTI by capturing how connections to the situated environment are related to teachers’ identity representations, and how such representations feature in our focal CFL teachers’ metaphors and explanations.

There is a voluminous literature on the influencing factors of LTI. Over the past two decades, most research has emphasized how LTI is mediated by the personal, institutional, and sociocultural contexts (Eslamdoost et al., 2019; Nazari et al., 2021). Relatedly, Aminifard et al. (2025) attributed a novice Iranian EFL teacher’s identity disillusionment to emotional labor. Yim and Mercer (2025) found that in-service English teachers’ identities have been undermined by the native-speaker ideology that permeated the South Korean society.

### 2.2 Understanding metaphor and elicited metaphor analysis

In their seminal work *Metaphors We Live By,* Lakoff and Johnson (1998) contended that “…metaphor is pervasive in everyday life, not just in language but in thought and action. Our ordinary conceptual system, in terms of which we both think and act, is fundamentally metaphorical in nature”. They also defined metaphor as “understanding and experiencing one kind of thing in terms of another” (p. 5). By analyzing how people talk about one thing (the target) in terms of something else (the source), we could understand how people think about one thing in terms of something else (Reijnierse and Burgers, 2023).

Basically, a metaphor consists of a source domain and a target domain. Fisher (2013) noted that researchers need to identify the association between the metaphor’s target domain (the topic) and the source domain (the vehicle). O’Reilly and Marsden (2021) explained that metaphor is described in terms of the topic conveyed (anger) and vehicle/words used (e.g., “fuming”) in language, whereas conceptually, the distinction is described as a target domain (ANGER) and a source domain (FIRE). The target domain is generally more abstract or complex such as “learning”, whereas the source domain is a more concrete concept or familiar image such as “a steep hill” (Geng et al., 2024; Jin et al., 2014).

Among the two approaches to metaphor analysis, the first approach requires collecting metaphors from analogical statements that arise naturally in conversation or writing, and the second approach, also known as Elicited Metaphor Analysis (EMA) involves a prompt completion task, i.e., an “A IS (like) B” structure (Wan, 2014; Wan et al., 2011). EMA researchers maintain that the entailment (Jin et al., 2014), or the because phrase (Saban et al., 2007) is indispensable. The entailment/ because phrase encourages participants to think of vehicle terms (e.g., in this study, entities to which a language teacher is compared) that carry insider’s evaluations, attitudes, values, perspectives and beliefs (Cameron and Maslen, 2010; Naghavian, 2024). It also prevents the imposition of outsider meaning (Cortazzi and Jin, 2020).

It is noteworthy that not all prompted responses are analyzable and valid, answers without metaphorical reasoning should be excluded (Wan, 2014). For instance, Low (2003) argued that Bartelt’s (1997) example of LANGUAGE LEARNING IS TRANSLATION cannot be considered a metaphor, because the link between learning and translation is quite literal or part-for-whole metonymy. Similarly, Burgers et al. (2016) noted that the source and target domain have to be remote enough so that the leap from source to target contains two distinct domains. To guide rigorous implementation of EMA, Low (2015) proposed a seven-step validation model, including training participants before data collection, providing tasks that allow one or more metaphors, illustrating a high-level conceptual or professional orientation, allowing participants to reevaluate whether their responses actually change, etc. (Wan and Low, 2015).

### 2.3 Metaphor as a lens for understanding language teacher identity

In teacher education, metaphors have communicative function because they embed knowledge, beliefs, and experiences relevant to developing teacher identity (Schellings et al., 2024). Rooted in people’s subjective experiences and imaginations, metaphors enable us to organize the multiple meaning constructions of self and reality into systematic concepts (Gao and Cui, 2021; Saban, 2006). By using an object or event as conceptual tools, metaphors reduce teachers’ complex educational philosophies and actions into comprehensible images (Derakhshan et al., 2023; Martínez et al., 2001). With metaphors, teachers could verbalize implicit and abstract constructs into concise and vivid language, and express “what otherwise would appear indescribable” (Craig, 2018; Thornbury, 1991).

In essence, teacher identity is a teacher’s subjective sense-making of who he/she is as a teacher. Given the complexity and multiplicity of teacher identity, and the functions of metaphor, we might safely conclude that metaphor could serve as a reflective tool for teachers to look for who they are (Farrell, 2016) and represent their professional lives (Leonardsen et al., 2022), because a large part of self-understanding is the search for appropriate personal metaphors that make sense of our lives (Lakoff and Johnson, 1998).

An abundance of literature has used metaphor to investigate LTI in diverse settings (Chien, 2020; Kamil, 2022; Liu, 2023; Xiong et al., 2015; Yim and Mercer, 2025; Zhu et al., 2020). One strand of research utilized longitudinal design to track teachers’ evolving identities during practicum or reform (Chien, 2020; Gao and Cui, 2021; Nagamine, 2012). By comparing participants’ written metaphors before and after the practicum, Zhu et al. (2020) concluded that student EFL teachers’ identities transformed, and their teaching orientations have changed from behavioristic to constructivist. Similarly, novice teachers’ identity perceptions shifted as they transitioned from teacher education or development programs to actual teaching practice (Seyri and Nazari, 2022; Trent, 2020). Another line of research compared teachers and students’ perspectives on teachers’ identities (Liu, 2023; Wan et al., 2011; Wood, 2025). The third line of inquiry delved into the influencing factors of LTI perceptions. Taken together, the factors that influence LTI perceptions include learner profiles (Kamil, 2022; Nguyen, 2016), institutional culture (Feng, 2024), and socio-cultural particularities (Yim and Mercer, 2025).

Concerning how language teachers metaphorically represent themselves, research findings have revealed a wide variety of metaphors, including travel agent, architect, construction foreperson, chef (Nagamine, 2012), director, cook, engineer, candle, entertainer (Xiong et al., 2015), artist, mother, trial judge, democrat, intercultural promoter (Nguyen, 2016), magician, sister (Chien, 2020), role model, friend, parent, facilitator, authority (Liu and Sammons, 2022), friend, manager (Wood, 2025). In a Chinese university, Zhang (2019) focused on 11 Less-commonly-taught Foreign Language teachers’ identities, and showed that they ranked their identities as teachers, communicators, and all-rounders.

In terms of the meanings of metaphors, most studies have reported positive metaphors, such as candle, engineer (Xiong et al., 2015), sailor, pilot, film director, actor (Liu, 2023), intercultural promoter (Nguyen, 2016), knowledge provider, nurturer, leader (Lin et al., 2012; Liu, 2010), and sun (Kamil, 2022). More recently, however, Lyu and Lam's (2025) review study on private tutor teacher identity indicated mixed metaphors, such as educators and service providers. Among the reviewed empirical studies, Xiong et al. (2022) reported that teachers from private tutoring institutions depict themselves as exam experts, salesperson, and underdogs. Wang et al. (2021) identified four negative metaphors (attendant, firefighter, coolie, tramp) to capture identity perceptions of one English teacher working at a tutoring institution.

## 3 Methodology

A qualitative research design was adopted, as it is suitable for exploring and understanding participants’ ascribed meanings to a certain phenomenon or problem (Fossey et al., 2002). Among the data collection methods for qualitative research, we used EMA, semi-structure interview protocol, and document analysis.

### 3.1 Research context and participants

From the universities we have access to, four universities that represent different rankings and distinctive institutional cultures were selected (Table 1). University A is a “double-first class university” in a second-tier city in North China, university B is one of the top five universities in an economically developed second-tier city in Southeast China’s coastal province. Teachers from Universities A and B face more pressure to be research-productive compared to their counterparts at C and D (Policy documents). The variations in terms of university orientation and regional development enable a more thorough and nuanced understanding of teachers’ working environment.

**Table 1:** Details of research contexts.

University	University type and location	University orientation
A	Public comprehensive university, Second-tier city	Teaching and Research-oriented
B	Public polytechnic university, Second-tier city	Research-oriented
C	Public teacher training college, Fourth-tier city	Teaching -oriented
D	Private college of finance and economics, Third-tier city	Market-oriented

We also targeted teachers with diverse background (gender, years of service, academic ranks), as this would make the sample representative enough of foreign language teachers in the Chinese higher education system. Participants were recruited through snowball sampling, initiated through our professional contacts with the four teachers working, respectively, at the above-mentioned universities. We relied on them to identify and recruit more participants. The criteria for participant selection are:

(a) both male and female teachers must be included;
(b) teachers with different years of experience should be included;
(c) at least one professor and four associate professors should be included from each university.

After a period of 3 months, 88 teachers agreed to participate. However, six of them could not recall any suitable metaphors upon repeated explanations, therefore valid answers from 82 teachers (75 English teachers, 2 Japanese teachers, 2 Russian teachers and 3 German teachers) were used for analysis. The socio-demographic information of the participants is presented in Table 2. To protect the participants’ anonymity, we label them as A1-A19, B1-B22, C1-C19, D1-D22. Prior to data collection, we thoroughly explained the study and gained written approval from each participant.

**Table 2:** Socio-demographic information of research participants (*N* = 82).

University	Gender		Degree		Academic rank			
	F	M	PhD	Master	Prof	Assoc. Prof	Lecturer	Teaching assistant
A (19)	14	5	2	17	1	4	14	0
B (22)	16	6	10	12	1	4	15	2
C (19)	15	4	4	15	1	4	14	0
D (22)	18	4	0	21	1	4	14	3
Total	62	20	16	66	4	16	57	5

### 3.2 Data collection

Data were collected from metaphor elicitation tasks, follow-up interviews, and documents. Following Low’s (2015) guidelines on rigorous implementation of EMA (Wan and Low, 2015), we explained what metaphor means before asking each participant to complete the task: As a university language teacher, I am/like a ____________because____________. The because phrase was added to facilitate the coding process and categorization, as the same metaphor is usually used to express diverse meanings (Fisher, 2013). Participants were informed that one or more metaphors were allowed.

After 1.5 months, we began the interview session with each participant. The time interval was designed on purpose for us to elicit possibly addition of alternative metaphors from the same participant. Although most participants were proficient in English, all of them indicated they felt more expressive at communicating ideas in their mother tongue. The English version of semi-structured interview protocol was attached as Appendix A in Supplementary material. Participants were asked to explain their metaphor(s) further and share their experiences regarding their teaching, and research experience. The interview data helps researchers confirm whether the collected metaphors are negative or positive. The interviews were conducted via WeChat (the most popular social media application in China), depending on the convenience of participants, and each interview lasted on average 40 min as conducted in Chinese.

Relevant documents, including teacher profiles, university policy documents, faculty website reportage on faculty teaching and research activities were also collected for verifying findings or corroborating evidence from other sources (Bowen, 2009). The institutional teacher recruitment and appraisal documents, with requirements for teachers’ research and teaching performance and details on indicator scores, can be used to triangulate the collected metaphors and interviews. Teachers’ profiles (age, gender, academic rank, teaching awards, research grants, research publications, etc) could supplement research data. Additionally, the website reportage enables us to better understand the differentiated faculty environment in which teachers were situated.

### 3.3 Data analysis

We used both inductive and deductive reasoning and the analysis procedure was an iterative process (Srivastava and Hopwood, 2009). Data analyses involved four stages: (1) checking the validity of teachers’ answers (whether they are metaphors or not); (2) ascertaining positive/negative meanings; (3) generating conceptual categories; (4) exploring factors that underlie the use of metaphors.

#### 3.3.1 Stage one

We eliminated invalid answers from further analysis. An example is the answer from a male lecturer: “As a university teacher, I feel I am a decent poor man because the job is decent but my income is meager” This answer was excluded because no metaphorical reasoning was detected (Wan et al., 2011), and the target domain and the source domain is not sufficiently remote (Burgers et al., 2016). Hence, 81 teachers’ answers were considered valid. As some participants used multiple metaphors to represent their identities, we used the + sign to indicate these two or three metaphors come from one participant (Appendix B in Supplementary material).

#### 3.3.2 Stage two

To answer the first research question, the “positive/negative” meaning of each metaphor was coded as per the meaning the participant ascribed to the metaphor(s). After reading teachers’ metaphors, complementary explanations, and the follow-up interview data repeatedly, we focused on the topic (i.e., the language teacher), the vehicle (i.e., the term to which language teacher is compared, such as a mother), and the nature of the relationship between the vehicle and the topic (Wan et al., 2011). For instance, the vehicle “bee” was used by five teachers, while four teachers associated the metaphor with negative meanings such as heavy workload and lack of work-life balance, one teacher used “bee” to indicate her hardworking attitude. For certain metaphors that arouse controversy, such as whether “assembly line worker” and “company employee” were neutral or negative, the researchers negotiated with each other several times until consensus was reached.

#### 3.3.3 Stage three

Thirdly, we grouped the linguistic metaphors into conceptual categories as per the affinity of meanings. For instance, the metaphors “scaffold”, “relay station”, “midwife”, “catalyst” were grouped into “FACILITATOR”, the metaphors “bee”, “old ox”, “snail”, and “camel” were categorized as “ANIMALS”. For ease of comparison, the conceptual category labels were further refined based on the relevant research findings. For instance, we drew on “LEADER”, “NURTURER”, and “INTERCULTURAL PROMOTER” from prior studies (Kamil, 2022; Liu, 2010; Nguyen, 2016).

#### 3.3.4 Stage four

Finally, to explore why CFL teachers used particular metaphors to represent themselves, we analyzed teachers’ metaphors and interview transcripts. The documentary data were analysed together with data from metaphor tasks and interviews, so that themes and sub-themes would emerge across all three sets of data (Bowen, 2009) (Table 3). For instance, we identified institutional demands as the factor that underlie teachers’ “academic laborer”, “camel”, “snail” metaphors. When analyzing “academic laborer” metaphor, we also cross-check the teachers’ academic profiles, and the institutional documents on teachers’ research output.

**Table 3:** A sampling of documents and data analyzed.

Source	Documents selected	Data analyzed
Teacher profile	Many teaching rewards and research publications	Evidence that teacher has high sense of self-efficacy
University B	Supplementary employment agreement for introduced talents Position setup and appointment objectives for full-time faculty in the school of foreign studies	University B imposes stringent research requirement for newly recruited PhDs and other staff
University B	Catalogue of performance indicators	Only high-level papers are recognized
Teacher profile	Teachers’ year of service, teachers’ student-related work	Evidence that teacher is passionate and experienced
University D	Professional title evaluation measures	Promotion criteria are lenient
University A, C	Faculty webpage reportage	A few research seminars
University D	Faculty webpage reportage	Almost no research seminars

To enhance the trustworthiness of the study, the written metaphors, accompanying explanations and interview transcripts were translated from Chinese into English by first author and sent to the co-authors for proofreading (all the three authors are high-level translator certificate holders) (Yunus et al., 2022). Concerning different ideas on how to translate certain Chinese culture-loaded metaphors (louyi VS mini-insect; corporate slave VS temporary worker; academic migrant worker VS academic laborer; a strong arrow at the end of its flight VS the last gasp of a powerful crossbow), additional advice was sought from a PhD in translation, one of the first author’s colleagues until a joint decision was reached (Appendix C in Supplementary material). The three authors coded separately and discussed the emerging themes each one of us identified. After several rounds of discussions about the codes and themes, eventually we achieved an inter-rater agreement of 91% (O’Connor and Joffe, 2020). We further solicited feedback from our participants and invited them to cross-check our interpretation of their data (Motulsky, 2021).

## 4 Findings

The findings will be presented under three sub-headings informed by the three research questions: 4.1 Meanings of metaphors, 4.2 Categories of metaphors, and 4.3 Contributing factors. Except for English teachers, teachers of other languages are marked in the brackets following participants’ quotes.

### 4.1 Meanings of metaphors

Among the 91 valid metaphors developed by 81 teachers (Appendix B in Supplementary material), two metaphors (island, dictionary) indicate neutral meanings, 51 are positive, and 35 are negative.

#### 4.1.1 Positive metaphors

As shown in Table 4, 51 metaphors carry positive meaning, and had a frequency ranging from 10 to 1. The positive metaphors with the highest frequency were: “lighthouse” 10 times, “bridge” 4 times, “gardener” 3 times, “actor” 3 times. Five of these positive metaphors (tourist guide, sun, satellite, pathfinder, navigator) have frequencies of two.

**Table 4:** The distribution of 51 positive metaphors.

Metaphor		F	Metaphor		F	Metaphor		F
1	Lighthouse	10	11	Tutor	1	21	Key	1
2	Bridge	4	12	Mentor	1	22	International tourist guide	1
3	Gardener	3	13	Midwife	1	23	Coach	1
4	Actor	3	14	Scaffold	1	24	Craftsman	1
5	Tourist guide	2	15	Relay station	1	25	Doctor	1
6	Sun	2	16	Catalyst	1	26	Flame	1
7	Satellite	2	17	Supporter	1	27	Candle	1
8	Pathfinder	2	18	Forest guard	1	28	Trader	1
9	Navigator	2	19	Soul engineer	1	29	Mother-to-be	1
10	Guide	1	20	Mother	1	30	Bee	1
Total		31	Total		10	Total		10

F, refers to frequency.

#### 4.1.2 Negative metaphors

Teachers have developed 35 negative metaphors (Table 5). The most frequently mentioned negative metaphors were bee (4); nanny (4), academic laborer (3), corporate slave (3), chicken rib (2), louyi (2). Seventeen metaphors other than these were expressed only once. In Chinese culture, “chicken rib” means good-for-nothing, “louyi” (literally means mole cricket and ant) symbolizes a tiny and insignificant being. Other images such as “old ox”, “camel”, and “snail” were mentioned as miserable lives leading a harsh life and pressing ahead with difficulty.

**Table 5:** The distribution of 35 negative metaphors.

Metaphor		F	Metaphor		F	Metaphor		F
1	Bee	4	9	Street vendor	1	17	A strong arrow at the end of its flight	1
2	Nanny	4	10	Company employee	1	18	Tool	1
3	Corporate slave	3	11	Old ox	1	19	Repeater	1
4	Academic laborer	3	12	Camel	1	20	Teaching machine	1
5	Louyi	2	13	Snail	1	21	Spinning top	1
6	Chicken rib	2	14	Eunuch	1	22	Lone ranger	1
7	Migrant worker	1	15	Customer service	1	23	Sandwich	1
8	Brick carrier	1	16	Servant	1			
Total		20	Total		8	Total		7

#### 4.1.3 Ambivalent metaphors

It is noteworthy that *B19 described herself as **a machine with emotions**. In addition,* a few CFL teachers ascribed positive meanings to the seemingly negative metaphors they produced:

*I am **a frontline worker**, it’s tiring, but I feel **satisfied** as a teacher. (B8).*

*I am just like **an assembly line worker** in the factory, producing batches of products continuously. Sometimes I feel tired and bored. But most importantly, **I want to be a better worker and process raw materials into better products** (I mean, cultivate better talents). (C15, interview).*

### 4.2 Categories of metaphors

As Table 6 displays, teachers producing positive metaphors generally identified themselves as GUIDES (tourist guide, guide, pathfinder, navigator, mentor, tutor), FACILITATORS (catalyst, midwife, scaffold, satellite), NURTURERS (forest guard, soul engineer), INTERCULTURAL PROMOTERS (bridge, key), and PROFESSIONALS (actor, doctor). Positive metaphors mostly evoked light images (lighthouse, candle, sun, flame), and human images (caregivers and professionals). Surprisingly, the “candle” metaphor, which was traditionally used to depict Chinese teachers occurred only once. Negative metaphors can be grouped into HARD WORKERS (migrant worker, street vendor, corporate slave), SERVICE PROVIDERS (nanny, eunuch, servant), LABORIOUS ANIMALS (bee, old ox, snail, camel), INSIGNIFICANT BEINGS (chick rib, louyi), and even INANIMATE OBJECTS (teaching machines, repeater).

**Table 6:** Categories of metaphors.

Category	N	Positive metaphor (51)
GUIDE	17	Lighthouse (10); pathfinder (2); tourist guide (2); guide; tutor; mentor
FACILITATOR	7	Satellite (2); midwife; scaffold; relay station; catalyst; supporter
NURTURER	6	Gardener (3); forest guard; soul engineer; mother
INTERCULTURAL PROMOTER	6	Key; bridge (4); international tourist guide
PROFESSIONAL	6	Actor (3); coach; craftsman; doctor
LIGHT EMITTER	4	Sun (2); flame; candle
LEADER	3	Navigator (2); trader
Other	2	Mother-to-be; bee

Category	N	Negative metaphor (35)
HARD WORKER	10	Corporate slave (3); academic laborer (3); migrant worker; brick carrier; street vendor; company employee
LABORIOUS ANIMAL	7	Bee (4); old ox; camel; snail
SERVICE PROVIDER	7	Nanny (4); eunuch; customer service; servant
PETTY BEING	5	Louyi (2); chick rib (2); a strong arrow at the end of its flight
INANIMATE OBJECT	4	Tool; repeater; teaching machine; spinning top;
OTHER	2	Lone ranger; sandwich

Category	N	Conflicting metaphors (3)
	3	Machine with emotions; frontline worker; assembly line worker

Category	N	Neutral metaphors (2)
	2	Dictionary; island

### 4.3 Contributing factors

Analysis of the interview data further reveals factors that underlie CFL teachers’ selection of certain metaphors. The factors can be categorized into teachers’ personal philosophies and self-efficacy, institutional demands, socio-cultural environment, students’ attitude, disciplinary status, and faculty support.

#### 4.3.1 Teachers’ personal philosophies and self-efficacy

The dominance of positive metaphors appears to be grounded in teachers’ diverse values, and beliefs regarding how English should be taught and learnt, as well as teachers’ and students’ respective responsibilities.

*I am like **a gardener** because I can see all kinds of grass and flowers in my garden. They need my careful cultivation to thrive. (A1).*

*I am like **a tourist guide**, I lead my students to explore the wonderland, I can answer some of their questions, but students should explore more wonders by themselves. (B10).*

*I am like the students’ **mother**, because I believe a teacher should educate the students morally. (B3).*

*I am like **a coach** that passes down my knowledge and methods. Students should practice on their own, I am responsible for observing, encouraging, and evaluating.* (C5).

*I am like **a doctor** that diagnoses students’ characteristics and offers different learning methods based on students’ characteristics. As university teachers, we should not impose same requirements on all the students, as middle school teachers did. Different students learn English for different purposes, they have different learning needs*. (C19).

*The teaching profession is noble and glorious, we are **soul engineer**s that not only teach knowledge but also cultivate souls. (D4).*

*I think I am like a **satellite** around the sun, because student is at the center, I communicate with my students, understand their needs, and help them in many ways. (D21).*

*I feel like an **actor** because I act differently before different types of students. You see, each student has his or her own characteristics…for those shy students and students with low self-esteem, I will not be very harsh or critical on their oral English performance, I act gently because I try to reduce their fear of speaking English in class and in public. But for students who lack self-discipline, I will not be so mild, I can be strict.(A17).*

*I feel like an **actor** because I act differently before different types of students. For example, when teaching high-achieving students, I play the role of* “the wise man in the drama”, *because I strive to stimulate students’ intrinsic motivation and challenge them to think and learn more deeply. When teaching low-achieving students, I act as a “loving mother” because step-by-step guidance and more patience are needed. (D13).*

Clearly, the above metaphors show that teachers endorse various teaching philosophies and enact diversifying practice: while the “tourist guide” (B10) and “coach” (C5) metaphors suggest the belief that students should take the major responsibility in learning English, the “satellite” (D21) metaphor implies more student-centeredness. These metaphors reveal that teachers from different levels of universities share similar beliefs: The “lighthouse” (A2, A14), “tourist guide” (B10), “coach” (C5), and “mentor” (D6) metaphors signify teachers’ affirmation of their roles in passing down knowledge and techniques. The “scaffold” (A12), “relay station” (A18), “catalyst” (A22), “midwife” (D20) metaphors indicate that teachers identify strongly with their role as a facilitator. The “mother” (B3) and “soul engineer” (D4) metaphors highlight teachers’ role in teaching moral values. Additionally, the “gardener” (A1), “doctor” (C19) and “actor” (A17, D13) metaphors demonstrate that teachers are fully aware of students’ individual differences A17’s putting on both “mild” and “strict” faces demonstrates his sensitivity to students’ negative emotions when speaking in public, as well as his ability to manage less disciplined students. Similarly, D13’s acting as a “wise man in the drama” and a “loving mother” captures her professional expertise.

It has been observed that while most teachers across four levels of universities exhibit high self-efficacy, some teachers also experience low self-efficacy, as evidenced by metaphors such as the chick rib (A3), a strong arrow at the end of its flight (B20), repeater (C10), and street vendor (D19). Furthermore, when teachers possess high level of self-efficacy, i.e., they affirm their ability to positively influence students’ lives in one way or another; they tend to present highly positive metaphors. This is evident in the following explanations from CFL teachers across the four different universities:

*I am **a lighthouse** because I provide direction and guidance for my students, I illuminate their academic journey and help them avoid hurdles, and encourage them towards their goals. (A14).*

*I am **a key** that enables my students to open the treasure house of language and culture. (B18, Russian teacher).*

*I am like **a navigator** that helps students find better flight routes. I lead my students towards the established goals by assigning them tasks and helping them improve during the tasks. (C1).*

In contrast, when CFL teachers’ self-efficacy is low, self-depreciating metaphors are provided. In Chinese culture, chicken rib originates from a historical anecdote, nowadays it refers to someone or something that has not much use.

*I am like **the chicken rib**. Competent students can do well without my help. No matter how much I tried, I cannot help all the students, no matter how much students trust me, they cannot learn English well without paying efforts. (A3).*

#### 4.3.2 Institutional demands

Other contributing factors relate to CFL teachers’ challenges in coping with the institutional demands, including heavy workload, job instability, and research output-driven policy. The workload challenge appears to be more acute in private universities where CFL teachers are often assigned additional workload.

*We as private university teachers have to deal with large class size (over 60 students). They (the university leaders) squeeze the students into fewer classes to save cost, they will not hire more teachers to spread our workload, so I am **corporate slave** here. (D2).*

*I am **a busy bee,** teaching is only part of my work, I have to prepare students for competitions, CET-4 and CET-6, thesis writing, postgraduate entrance exam, even some administrative duties are assigned to us. (D7).*

*I am a **teaching machine** that repeats the same job year after year, I cannot see the value and meaning in my job, I have many class hours. (D15).*

*I am busy with preparing for and giving lectures, answering students’ questions, correcting homework, etc. So I feel like **a bee.** (D16)*

*We are assigned all kinds of work in private universities, I feel like **a bee.** (D22)*

Similarly, teachers from public universities also described themselves in a degraded manner:

*Like **a bee**, I can hardly draw a line between work and life. With the assigned research tasks, I have to work during summer and winter vacations. (B16, German teacher)*

*As the only Japanese teacher in this college, I have too many classes to teach, almost 30 classes per week, so I feel like a very tired **old ox.** (C14, Japanese teacher)*

Additionally, while public university teachers are entitled *Bianzhi* (a Chinese cadre system that ensures permanent position and better welfare), private university teachers work on a contractual basis, the unstable nature of the job position affects CFL teachers’ identity perceptions. Consider the following examples:

*My identity is a **corporate slave**, we are assigned extra work but no corresponding compensation. We are also facing the risk of getting fired some day.* (D3)

*Public university is the best workplace to go for, we as private university teachers risk losing our jobs. We are **corporate slaves**.* (D13)

Different from other universities, University B, in its vision to join the *Double First-Class University* list has enforced formidable tasks on CFL teachers: newly recruited PhDs are required to secure two provincial-level research grants, or one ministry-level research grant (or participate in national-level research program), publish a book and three key journals within 5 years in order to pass the appraisal. For newly-recruited associate professors, the tasks are doubled. Associate professors and professors will be demoted to lower academic ranks if they fail in the four-year performance appraisal (*policy documents*). Such research-oriented policy stood out as a prominent factor that influences teachers’ identity construction. In this respect, two newly graduated PhD said:

*With the research-related tasks, I cannot afford much time for relaxation during weekends or holidays. I feel like **a snail with a heavy shell on its back**. (B11)*

*I feel like **a camel struggling in the desert**. I am under great pressure to publish quality papers and obtain high-level research grants. The chances of obtaining state-level grant is so slim. I also have to guide students’ thesis, and as a new teacher, I must attend the teacher training courses before I can teach … (B17)*

The above-mentioned policy enactment has also led other teachers to develop negative metaphors:

*I feel like **an academic laborer** because my devotion and return is severely disproportionate. (B10)*

*I feel like **a louyi**, we as teachers do not have any say in neither assessment criteria nor the journal authorship recognition. Publications in some high-quality ESCI journals are not recognized. They only recognize SSCI, CSSCI. Corresponding authorship through cross-university cooperation is also denied credit. (B12)*

*I am a **louyi**, as I have no say in policy enactment. Currently, we must contribute to the university ranking, just like **company employees** must contribute to the company GDP. (B14)*

*University teachers are literally **academic laborers.** The salary is meager. It is difficult to obtain research grants, publishing papers is equally hard. The competition is too fierce. (B16)*

#### 4.3.3 Sociocultural environment

Sociocultural environment refers to the marketisation of education, and the perceived declining social aspect for teachers. Traditionally, teachers are held in high regard in Chinese society, surprisingly, however, many teachers depicted themselves as service providers. See, for example:

*I am a **nanny**, because I have to sort out learning problems for them. They (the students) are my bosses, because they can throw numerous weird questions to me. (C7, interview)*

*Nowadays, the social respect for teachers is declining. People worship money instead of knowledge. So I feel like **a nanny** that provides service for my students. (A10, interview)*

*I am like a **customer service**, like the **emperor’s eunuch**, because I need to take responsibility, abide by the professional guidelines, and improve my service quality. (A8, interview)*

In Chinese culture, a nanny, a hired employee at home to take care of the baby or the elderly, and the household chores has low social status. A eunuch is a very close and dedicated servant who takes charge of all the daily activities for the emperor in the Chinese feudal society. Clearly, all these CFL teachers positioned their status as lower than their students.

#### 4.3.4 Students’ attitude

Students’ attitude emerges as another influencing factor that affects CFL teachers’ identity perceptions (A3, B19, C3, D8, D19). Teachers across four universities concur that who they feel/think they are were directly shaped by students’ attitude towards learning, as they expressed:

*I am **a teaching machine with emotion**s, and my emotions fluctuate based on students’ interactions. In some classes, I taste the joy of teaching, but in classes where students never respond, I become slack at work and feel they are not worthy of my emotions to this course. (B19)*

*I am like **a dictionary**, because some students can obtain wealth of knowledge, while other students only use the dictionary as a table mat. (C3)*

*I am like **an island**, those who love learning can find fortune, while those who pursue nothing more than a certificate only travels here. (D8)*

*I feel like **a street vendor.** Many students played cellphones in class, ignoring me on stage. They only look at me whenever I say something interesting. But they quickly lower their head and play their cellphone! No matter how hard I promote my products, I feel my students feel I am coaxing them into buying the products! I think English should be taught only to students who have interest, in this way, I won’t be so tired! (D19)*

*In addition, students’ attitude towards teachers also influences how teacher portrays her identity:*

*I feel I am a **tool** for my students, they never come to me until they fail the exam, nowadays, the students have utilitarian mindset. (C8)*

#### 4.3.5 Disciplinary status

Two of these teachers’ metaphors could be attributed to the declining status of the language discipline:

*In our university, the leaders attach importance to the engineering disciplines and pay little attention to the language subjects. English is a marginalized discipline that serves other disciplines, so I feel I am **a servant,** just like the servants in our university canteen. (B9)*

*The subject English has passed its prime, with the development of AI and the availability of English learning materials, teachers’ authority has been weakened, so I feel like **a strong arrow at the end of its flight**. (B20).*

#### 4.3.6 Faculty support

Furthermore, the university website reportage shows that faculty-level research-related activities (expert lectures; sharing sessions on teaching and research) are more frequently held in University B than University A and C. Due to economic constraints, very few research-related activities are held in University D. C6’s metaphor was associated with the faculty support mechanism. As he reflected:

*I feel like **a lone ranger**, I have to give lectures and do research all by myself, I have to grope in the dark all by myself, nobody guides m*e. (C6, interview, German teacher)

Similarly, when asked about the faculty environment, A10 lamented on the lack of research atmosphere in his faculty, he said:

*Although we work in a Double First-class University, very few language research-related seminars are held at faculty level, most of my colleagues are holding master’s degrees, and we do not know how to do research, so many adopted a lying-flat attitude to academic research.* (A10, interview)

#### 4.3.7 Interplay of multiple factors

CFL teachers’ mixed or conflicting identity perceptions can be interpreted in light of the multiple factors that influence identity perceptions. For instance, although A10 developed the “nanny” metaphor, he also affirmed himself as a “lighthouse” that illuminates his students’ road:

*I think I am like **the lighthouse**, because I strive to illuminate students’ future road. I hope I can help them to become postgraduate student or find ideal jobs.(A10).*

In another case, a teaching assistant’s “sandwich” metaphor showcased the multiple influence of national (national guidelines on English teaching, national reform on reduced English teaching hours), institutional (teaching syllabus mandate), and personal factors (teachers’ teaching belief) on LTI. As she expressed:

*I am like **a sandwich** in between, the upper part is top-down mandate, including the teaching syllabus at university level and the national-level requirement, such as College English Teaching Guide issued by the Ministry of Education. The lower part is the students’ real needs and requirement of the job market. I feel the upper and lower parts are disconnected. I want to pass the knowledge, clarify the students’ doubts and cultivate students’ humanistic literacy, but students only want to learn the skills that help them pass CET-4 and CET-6. I have to follow the teaching syllabus, due to the College English class hour reduction policy, I have to complete my teaching tasks in very limited time, sparing little time to train exam-related skills. (C8)*

## 5 Discussion

**Figure 1:**
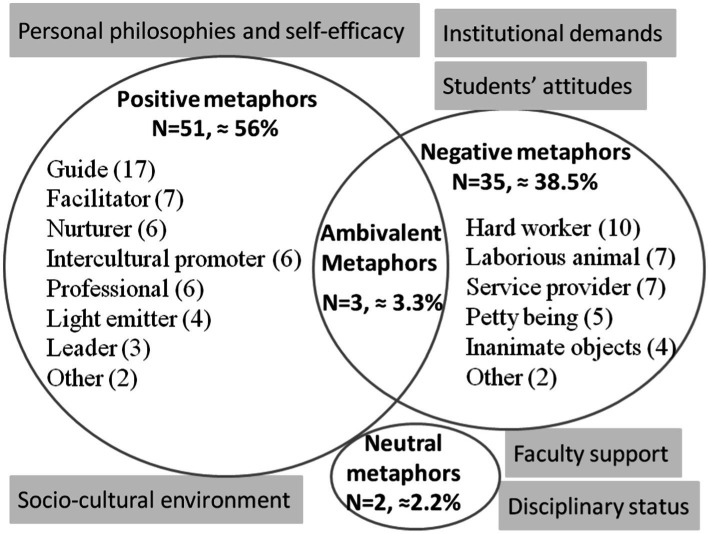
Patterns and influencing factors of CFL teachers’ metaphors.

Data analyses reveal the meanings and categories of CFL teachers’ metaphors (Figure 1). The study shows that CFL teachers’ identity perceptions encompass personal, interpersonal, institutional, and socio-cultural dimensions, they develop and change their identities under situated environment, subsequently integrating the contextual influences into their own qualities in the internalization process (Johnson and Golombek, 2011).

As seen in Figure 1, of all the 91 valid metaphors, positive metaphors (N = 51) account for about 56%. Echoing prior findings, this study shows similar categories, including LEADER (Eren and Tekinarslan, 2013), FACILITATOR (Xiong et al., 2015), and NURTURER (Lin et al., 2012). The metaphors developed by Russian teacher (key), Japanese teacher (bridge), and Chinese teacher (bridge) highlight language teachers’ unique roles in imparting both linguistic and cultural knowledge, which resonate with the works of Zhang (2019), Xiong et al. (2015), and Nguyen (2016) in Chinese and Vietnamese context Concurring prior findings, findings also reveal culture-bound metaphors, including the LIGHT EMITTER metaphor which reflects the perceived teacher’s responsibility to “generate light and heat”, and the MOTHER metaphor grounded in the belief that a teacher plays a role as a parent in Chinese culture.

While positive metaphors abound in previous studies (de Laurentiis Brandao, 2021; Kamil, 2022), negative metaphors (N = 35) account for about 38.5% in this study. Surprisingly, many teachers compare themselves to “assembly worker”, “brick carrier”, or various animals, which deviate from teachers’ identities as intellectuals. Different from previous studies that identified SERVICE PROVIDER metaphors mainly in private tutoring institutions (Lyu and Lam, 2025), this study found that both public and private university teachers depict themselves as SERVICE PROVIDERs rather than KNOWLEDGE and TOOL PROVIDERS (Lin et al., 2012). These findings could be explained by self-determination theory (Deci and Ryan, 2012), which clarifies teachers’ basic psychological needs for competence (feeling achievable and capable), relatedness (feeling warm and connected), and autonomy (feeling having choices, and a sense of ownership) (Chiu et al., 2024). Teachers perceive themselves as “chick ribs”, “street vendors”, “snails”, “camels” when they feel not very competent in teaching, student management, or research, their “servant”, “nanny” and “tool” metaphors imply the unmet needs for relatedness, they feel like “company employees”, “louyi”, and “corporate slaves” because their needs for autonomy (the sense of control over their professional lives) are not satisfied.

The study further unravels a range of contributing factors to CFL teachers’ identity perceptions, including personal, interpersonal, institutional, and socio-cultural factors: On a personal note, some participants produce “coach”, “craftsman”, “mentor”, “doctor” metaphors with a strong emphasis on their professional expertise and practice. The “satellite” metaphor suggests a student-oriented teaching style, whereas the “trader” and “navigator” metaphor asserts the leading role of the teacher. Certain self-depreciating metaphors such as “repeater”, and “teaching machine” might be explained by the fact that irrespective of the field of study, each college student should take at least one foreign language course, hence CFL teachers are usually assigned more teaching hours compared to their counterparts teaching other courses.

At interpersonal level, the neutral metaphors “dictionary”, “island”, “teacher with emotions” are obviously shaped by students’ attitudes towards learning. Another interesting finding is that teacher-student relationship impacts teacher’s identity perception. When C8 noticed her students never came to her until they failed exams, she used “tool” to capture her identity.

At institutional level, quite a number of teachers, especially teachers from university B liken themselves to “academic laborer”, “bee”, “camel”, “louyi”, and “snail”, indicating the struggles and negative emotions CFL teachers experience in navigating their teacher-researcher nexus (Gong et al., 2024). One plausible reason is the low proportions of PhDs among CFL teachers, most CFL teachers are at a disadvantage in research publications (Lu and Yoon, 2022).

The “bee” and “corporate slave” metaphors offered by private university teachers point to the differentiated resource allocations among universities posed by the stratified HE system. This study further reveals that variations exist not only among institutions but also among different departments and disciplines within the same university, with B9’s “servant” metaphor mirroring the fact that less resources were allocated to English at a Polytechnic University. In addition, B14’s “company employee” and “louyi” metaphors relate to university authorities’ strategic planning within the stratified HE system (Hu et al., 2017). In China context, universities rely on governmental funding and reputation endowed by officially recognised classification schemes (Han et al., 2023). According to Bourdieu’s field theory, the HE is a social field where universities strives to improve their position by acquiring symbolic capital, which is akin to social recognition and reputation (Gerhards et al., 2018). Symbolic capital is most vital for universities as it directly determines the upward and downward mobility (Li, 2019), to obtain more symbolic capital and maintain a favorable position in the HE hierarchy, universities implement policies and rules that reward research excellence, hence teachers have to “sell” their intelligent labor to increase the competitiveness of their institutions in evaluations (Zhang et al., 2024).

Another finding is that some CFL teachers take on mixed identity perceptions. This is in line with previous research results (Yuan et al., 2020). A case in point is D5, a Japanese teacher who believes she acts as a “bridge” for students to know Japan and Japanese culture. She also feels like a “nanny” trying very hard to feed the students who are unwilling to eat (learn). Meanwhile, she compares herself to a busy “bee” dealing with all kinds of chores at a private university.

## 6 Conclusion and implications

By using metaphor as a lens, this study delves into the patterns and contributing factors of CFL teachers’ identity perceptions, and confirms that teacher identity perceptions are subject to the interplay of multiple factors at classroom, interpersonal, institutional, and sociocultural levels, we argue that stakeholders’ actions at all levels are essential for improving teachers’ identity perceptions. Findings of this study offer four major implications.

First, teacher education programs could inform prospective teachers of the challenges they are likely to face in an era of uncertainties so as to facilitate their adaptation and continuous professional development. In light of the negative metaphors (“camel”, “snail”, “academic laborer”) that mirror CFL teachers’ struggles against the assigned research tasks, we suggest that research literacy courses be incorporated into the teacher education programs to better prepare student teachers for the research demands. Prospective teachers should also be cognizant of the realities in classrooms. The student-related negative metaphors (such as “nanny”, “eunuch”, “street vendor”) not only reflect the permeation of commercialization, but also highlight the need for teachers to excel in student management. Teacher educators could host workshops and share strategies of dealing with unmotivated students.

Second, the “chicken rib”, “strong arrow at the end of its flight”, and “repeater” metaphors reveal some teachers’ identity crisis issue and warrant special attention. It is crucial for language teachers themselves to identify with their subject discipline and recognize their significant role in shaping the English teaching enterprise of the whole nation. Teacher educators may introduce them some innovative teaching methods, in so doing, teachers’ identity perceptions might shift and the negative self-portrays as “teaching machine”, “street vendor”, and “repeater” may be reduced. Besides, faculty leaders could organize sharing sessions and bring about alternative ideas and practices to them. They are also encouraged to proactively pursue lifelong learning and constantly improve their expertise.

Third, the “servant”, “louyi”, and “corporate slave” metaphors warrant actions from policymakers. Since identification with the subject discipline is an essential component of teacher identity, for language teachers to enhance their identity perceptions, the disciplinary bias towards language subjects at institutional level should be eliminated. If this is not dealt with, it causes B9 to feel like a “servant” working to serve other subjects. In addition, in light of the “louyi” metaphor that carrys the bitter and powerless feeling, the unilateral and rigid policy that denies authorship recognition, and the corporate management style have positioned teachers in a vulnerable and powerless condition. We call on policymakers to listen to teachers’ voices and weigh their policies against teachers’ responses when implementing novice demands.

Last but not least, given teachers’ perceptions are subject to the sociocultural environment, despite the marketisation of education, we argue that teachers should always be highly respected and not be left alone to self-care. We call for the whole society to rebuild a milieu in which teachers feel revered.

Our study suggests avenues for further research that could lead to a fuller understanding of CFL teachers’ identities. First, although our sample involved teachers at different stages of their career trajectories, and universities occupying different positions in the HE system, we did not tap into teachers’ identity and metaphor among those working at first-tier cities or Project 985 and Project 211 universities. This could be a research avenue for future researchers. Secondly, the study was conducted in a cross-sectional manner, longitudinal research into teachers’ identity perception over time could generate more insights into this agenda. Thirdly, the data sources are limited to metaphor elicitation tasks, interviews and documents, future researchers could use other data resources such as multimodal metaphors.

## Data Availability

The raw data supporting the conclusions of this article will be made available by the authors, without undue reservation.
