# Supplementary material for: “I am like a camel struggling in the desert”: understanding Chinese foreign language teachers’ perceived identities through metaphors
Source: Front Psychol. 2026 Mar 16;17:1696296. doi: 10.3389/fpsyg.2026.1696296 (PMC13034477; doi:10.3389/fpsyg.2026.1696296)
Supplement: Supplementary file 1 [file Data_Sheet_1.docx]

**Appendix A. Interview protocol**

1. In the metaphor form, you depicted yourself as____, has the metaphor changed along your career trajectory? If yes, what has caused the changes?

2. Could you please talk about your teaching in the classroom? How are your students?

3. Would you like to talk about your research experience?

4. What do you think of your university-level promotion and teacher assessment policy?

5. How do you evaluate your faculty working environment? (concerning colleagues, leaders, peer teachers)?

**Appendix B. Overview of valid metaphors (N=91) from CFL teachers (N=81).**

|  | Number of metaphors | Metaphors and frequencies |
| --- | --- | --- |
| A 1-19 | 23 | Lighthouse (7); gardener (3); candle; scaffold; customer service + eunuch; pathfinder + tourist guide +relay station; lighthouse+ nanny; chicken rib；actor; catalyst; craftsman |
| B1-21 | 23 | Lighthouse (2); guide；supporter; tourist guide; bridge; a strong arrow at the end of its flight; mother; satisfied frontline worker; servant; academic laborer; camel; snail; louyi; actor; company employee + louyi; spinning top; academic laborer + bee; key; machine with emotions; tutor |
| C 1-19 | 22 | Trader; navigator; flame; lone ranger; nanny+ academic laborer; coach; dictionary; tool + sandwich; repeater; chicken rib; bridge;  old ox; assembly line worker; bridge+ pathfinder; forest guard; doctor; sun + bee; satellite; |
| D1-22 | 23 | Corporate slave (3); soul engineer; island; actor; bee; mentor; sun; bridge+ bee+ nanny; teaching machine; migrant worker; mother-to-be; international tourist guide；brick carrier; bee; midwife; navigator; satellite; street vendor; nanny |

**Appendix C. The Chinese equivalent for the English translation**

|  | The English translation of culture-loaded metaphors | Raw data |
| --- | --- | --- |
| 1 | relay station | 中继站 |
| 2 | navigator | 领航员 |
| 3 | trader | 操盘手 |
| 4 | chick rib | 鸡肋 |
| 5 | corporate slave | 打工人 |
| 6 | louyi | 蝼蚁 |
| 7 | migrant worker | 农民工 |
| 8 | brick carrier | 搬砖工 |
| 9 | academic laborer | 学术民工 |
| 10 | a strong arrow at the end of its flight | 强弩之末 |
| 11 | lone ranger | 独行侠 |
